# Neonatal vitamin A injection promotes cattle muscle growth and increases oxidative muscle fibers

**DOI:** 10.1186/s40104-018-0296-3

**Published:** 2018-11-15

**Authors:** Bo Wang, Wei Nie, Xing Fu, Jeanene M de Avila, Yannan Ma, Mei-Jun Zhu, Martin Maquivar, Steven M Parish, Jan R Busboom, Mark L Nelson, Min Du

**Affiliations:** 1 State Key Lab of Animal Nutrition, College of Animal Science and Technology, China Agricultural University, Beijing, 100193 People’s Republic of China; 2 Department of Animal Sciences, Washington State University, Pullman, WA 99164 USA; 3 Department of Animal Sciences, Louisiana State University, Baton Rouge, LA 70803 USA; 4 College of Animal Science and Technology, Gansu Agricultural University, Lanzhou, 730070 Gansu People’s Republic of China; 5 School of Food Science, Washington State University, Pullman, WA 99164 USA; 6 College of Veterinary Science, Washington State University, Pullman, WA 99164 USA

**Keywords:** Cattle, Muscle Fiber type, Muscle growth, Myogenesis, PAX7, Satellite cells, Vitamin A

## Abstract

**Background:**

Vitamin A and its metabolite, retinoic acid (RA), are important regulators of cell differentiation and organ morphogenesis. Its impact on beef cattle muscle growth remains undefined.

**Method:**

Angus steer calves were administrated with 0 (control) or 150,000 IU vitamin A (retinyl palmitate in glycerol, i.m.) per calf at birth and 1 month of age. At 2 months of age, a biopsy of the *Biceps femoris* muscle was obtained to analyze the immediate effects of vitamin A injection on myogenic capacity of muscle cells. The resulting steers were harvested at 14 months of age.

**Results:**

Vitamin A administration increased cattle growth at 2 months. At 2 months of age, Vitamin A increased PAX7 positive satellite cells and the expression of myogenic marker genes including *PAX7*, *MYF5*, *MYOD* and *MYOG*. Muscle derived mononuclear cells were further isolated and induced myogenesis in vitro. More myotubes and a higher degree of myogenesis was observed in vitamin A groups. Consistently, vitamin A increased *Latissimus dorsi* (LD) muscle fiber size at harvest. In addition, vitamin A increased the ratio of oxidative type I and type IIA fibers and reduced the glycolic type IIX fibers. Furthermore, we found that RA, a key bioactive metabolite of vitamin A, activated *PPARGC1A* promoter, which explains the upregulated expression of *PPARGC1A* in skeletal muscle.

**Conclusion:**

Vitamin A administration to neonatal calves enhanced postnatal muscle growth by promoting myogenesis and increasing satellite cell density, accompanied with a shift to oxidative muscle fibers.

## Background

Muscle growth is due to both formation of muscle fibers and increase in muscle diameters. During embryonic muscle development, mononucleate myoblasts fuse to form primary myotubes [1]. Secondary myotubes form near the midpoint of primary myotubes under the basal lamina, then grow longitudinally, and eventually separate from the primary myotube [2]. It is widely believed that the total number of muscle fibers in a given muscle is fixed at or near birth for mammals [3, 4]. Postnatal muscle growth is achieved mainly by hypertrophy of existing myofibers [5], during which satellite cells proliferate and fuse with existing muscle fibers [6]. Thus, satellite cell abundance in postnatal muscle is closely associated with postnatal skeletal muscle development.

Skeletal muscle is composed of different types of fibers. Type I is a slow-twitch, oxidative fiber. Type IIA is a fast oxidative-glycolytic fiber. Type IIX is a fast-twitch, glycolytic fiber for beef cattle [7, 8]. The muscle fiber-type composition is an important factor in determining meat quality. Glycolytic muscle fibers accumulate greater amount of lactate during the postmortem stage [9], which is negatively associated with water holding capacity [10]. Muscles with high proportion of type IIX fibers had high lactate content and showed low muscle pH_45min_ [11]. Peroxisome-proliferator-activated receptor-γ coactivator-1 (PGC-1α) is a transcriptional co-activator abundant in skeletal muscle, which activates mitochondrial biogenesis and oxidative metabolism [12, 13]. PGC-1α activates calcineurin signaling and drives the formation of type I oxidative fibers [14]. Consistently, Vitamin A promotes mitochondriogenesis during brown/beige adipogenesis via activation of PGC-1α [15–17]. RA enhances PGC-1α and mitochondrial function in liver [18]. These data prompted us to further hypothesize that vitamin A promotes the shifting to oxidative muscle fibers in beef cattle. Up to now, the effects of nutrients on muscle fiber type composition in livestock remain poorly studied.

Vitamin A plays critical roles in animal growth and development. As an active metabolite of vitamin A, retinoic acid (RA) serves as a ligand for RA receptors (RAR) which partner with retinoid X receptors (RXR) [19]. The liganded RAR/RXR complex binds to retinoic acid response elements (RAREs) on target genes [20], which regulates gene expression. RA is an important morphogen during embryogenesis [21], which regulates cell differentiation including adipogenesis [22], myogenesis [23] and neurogenesis [24, 25]. Although several studies found that RA activates myogenesis in cultured cells and in rodents [26, 27], the role of vitamin A in muscle growth of beef cattle remain unexamined. Our previously study showed that administration of vitamin A at birth increased weaning weight of beef cattle [28], and the aim of this study was to explore the effects of neonatal vitamin A administration on bovine muscle growth and muscle fiber type composition.

## Methods

### Animal treatment

Animal studies were conducted at Washington State University Beef Center and Cattle Feeding Laboratory according to protocols approved by the Institutional Animal Care and Use Committee (IACUC). A total of twenty Black Angus steer calves were randomly selected during spring calving from an Angus based population of cows and heifers at the Washington State University Ensminger Beef Center. The calves were randomly separated into three groups injected (i. m.) with 0 (control), 150,000, or 300,000 IU vitamin A (retinyl palmitate in glycerol) at birth and 1 month of age. The detailed experimental design and animal performance data have been reported in another manuscript [28]. To facilitate biochemical analyses, in this study, we analyzed the difference in muscle fiber characteristics and myogenic potential of muscle tissue/cells between control and the 150, 000 IU vitamin A treated group (samples from 9 animals in each treatment were used, *n* = 9). The calves were weaned at 210 d of age and transported to the feedlot, where they were fed a backgrounding diet (50% steam-rolled corn, 30% grass hay, 15% potato co-products and 5% dry supplement) with free-choice trace mineral salt (98% NaCl, 0.509% Se, 0.006% Co, 0.01% I, 0.035% Cu, 0.20% Fe, 0.18% Mn, 0.037% Mg, 0.35% Zn) for 80 d. Then, cattle were transited to a finisher diet comprised of 59.5% steam rolled corn, 24% potato co-products (20% potato pieces, 4% cooked French fries), 8% grass hay, 5% dry supplement and 3.5% yellow grease. All diets were formulated to meet National Research Council (NRC, 2016) nutrient requirements for beef cattle. Nutrient analysis of the grass hay and final finisher can be found in Table 1, and composition of the dry supplement is listed in Table 2. Diet samples were collected as a weekly composite and analyzed by near-infrared spectroscopy (NIR). At 309 d of age, steers were implanted with Component TE-IS with Tylan (Elanco, Greenfield IL). Steers were harvested at the Washington State University Meats Laboratory at an average of 436 d of age. Weaning weight and weight gain during the backgrounding phase were increased by vitamin A injection [28].

**Table 1:** Nutrient analysis of grass hay and final finisher on DM basis

Component	Grass hay	Final finisher
Crude protein, %	6.4	13.1
Available protein, %	5.8	13.1
ADF^a^, %	35.2	7.2
aNDF^b^, %	56.5	11.4
Lignin, %	4.8	2.0
NFC^c^, %	28.7	63.3
Starch, %	1.1	55.1
Crude fat, %	2.3	6.6
Ash, %	6.08	5.60
TDN^d^, %	61	85
NEm, Mcal/lb.	0.57	0.98
NEg, Mcal/lb.	0.32	0.67
Ca, %	0.27	–
P, %	0.11	–

^a^ADF: Acid detergent fiber

^b^aNDF: Amylase-treated neutral detergent fiber

^c^NFC: Nonfiber carbohydrates

^d^TDN: Total digestible nutrients

**Table 2:** Composition of the dry supplement

Nutrient	Amount (DM)
Crude protein	76.9%
Crude fat (3.5% yellow grease on DM basis)	1.9%
Ca	12.31%
P	1.03%
Na	4.09%
K	0.3%
Mg	2.08%
S	0.24%
Mn	0.0615%
Zn	0.0924%
Cu	0.0308%
Co	0.0026%
I	0.0087%
Se	0.0006–0.0008%
Vitamin A	90,200 IU/kg
Vitamin D	9020 IU/kg
Vitamin E	226 IU/kg
Rumensin H	616 g/t
Tylan H	123 g/t

At 2 months of age, muscle biopsy (about 3 g) was obtained from the *Biceps femoris* muscle of each calf, as previously described [29]. Each muscle tissue sample was divided into 4 pieces. One piece (> 0.5 g) was put into cold PBS for satellite cell separation, one (> 0.5 g) was snap frozen in liquid nitrogen for RNA and protein extraction, one (> 0.5 g) was processed for cryosection, and the remaining one (> 0.5 g) was fixed in 4% paraformaldehyde for histological analysis.

### Muscle derived mononuclear cell isolation and myogenic differentiation

Biopsy muscle tissues were cut into small pieces and digested in a digestion buffer containing 0.75 IU/mL collagenase D (Roche, Pleasanton, CA) and 1.0 IU/mL Dipase type II (Roche) for 30 min at 37 °C. The lysate was filtered sequentially through 100 and 40 μm cell strainers, then centrifuged for 5 min at 500×*g*. The precipitated cells were then resuspended and seeded into collagen pre-coated 12-well culture plates at the density of 1 × 10^5^ cells/well. The resulting cells, containing myogenic satellite cells and nonmyogenic stromal vascular cells, were cultured in DMEM with 10% FBS for 2 d to reach 100% confluence. Myogenesis was induced in DMEM with 2% horse serum. The fusion index was calculated as the ratio of the number of nuclei inside myotubes to the number of total nuclei [30]. PBS and DMEM used in this study were supplemented with 100 IU/mL penicillin (Gibco, Grand Island, NY), 100 μg/mL streptomycin (Gibco), and 250 ng/mL Fungizone B (Gibco). After 6 d of differentiation, cells were fixed in cold methanol for 10 min, permeabilized with 0.1% Triton X-100 for 5 min, blocked with 1% BSA, and incubated with primary antibodies (anti-MHC 1:50; anti-desmin 1:100; Developmental Studies Hybridoma Bank, Iowa City, IA) at 4 °C overnight. Cells were then stained with corresponding secondary antibodies (1:1,000) at room temperature for 1 h, followed by incubation with DAPI for 10 min. Cells were viewed under an EVOS fluorescence microscope (10 images per animal).

### Tissue processing and histology

For analyzing muscle fiber structure and size, tissues were fixed in 4% paraformaldehyde (PFA) for 12 h at 4 °C, embedded in paraffin and sectioned (5 μm thickness). Sections were stained with hematoxylin-eosin as previously described [4]. The size and number of muscle fibers (at least 2,000 fibers per animal) were measured (6 images per section and 5 sections at 50 mm intervals per sample) using Image J (NIH).

For immunohistochemical staining, muscle tissues were frozen in isopentane cooled in liquid nitrogen. Frozen tissues were sectioned (10 μm thickness). Tissue sections were blocked with 5% goat serum in TBS containing 0.3% Triton X-100 for 1 h, incubated with anti-Pax7 antibody (1:10, Developmental Studies Hybridoma Bank, Iowa City, IA) overnight at 4 °C and the corresponding fluorescent secondary antibody for 1 h at room temperature. Sections were then mounted in a fluoroshield mounting medium with DAPI (ab104139, Cambridge, MA) and viewed under an EVOS fluorescence microscope (6 images per section and 3 samples at 100 mm intervals per sample).

Unfixed frozen tissue sections were used for muscle fiber typing by immunostaining of fiber-type specific myosin heavy chains. For type I fibers, BA-F8 (primary, IgG2b, 1:50; Developmental Studies Hybridoma Bank, Iowa City, IA) and Goat anti-mouse IgG2b Alexa488 (secondary 1:1,000) antibodies were used. For type I and IIa, BF-35 (primary, IgG1, 1:50; Developmental Studies Hybridoma Bank, Iowa City, IA) and Goat anti-mouse IgG1 Alexa555 (secondary, 1:1,000) antibodies were used.

### Plasmid transfection and luciferase

*PPARGC1A* promoter plasmid (#8887) was purchased from Addgene (Cambridge, MA). The plasmid was delivered to cells using Lipofectamin 3000 Reagent (Cat. No. L3000015, Thermo Fisher Scientific, Waltham, MA). The cells were treated with 1 μmol/L RA for 4 h and, then, the luciferase activity was measured using the Dual-Luciferase Reporter Assay System (Promega, Cat. No. E1910).

### Quantitative real-time PCR (qRT-PCR)

Total RNA was extracted from muscle tissue samples (0.1 g per steer) or cultured cells (8 × 10^4^ cells) with TRIzol reagent, followed by DNase (NEB, Ipswich, MA) treatment to remove DNA. The cDNA was synthesized using an iScriptTM cDNA synthesis kit (Bio-Rad, Hercules, CA). qRT-PCR was performed using a CFX RT-PCR detection system (Bio-Rad) with a SYBR green RT-PCR kit from Bio-Rad (Hercules, CA). Primer sequences are listed in Table 3. The cycling conditions comprised 2 min polymerase activation at 95 °C and 40 cycles at 95 °C for 15 s and 60 °C for 30 s. All PCR efficiencies were above 96%. Relative expression of mRNA was determined after normalization to 18S rRNA using the ΔΔ-Ct method. The gene expression was presented as fold changes to that of the control group. The validity of 18S rRNA as a reference gene was further verified using glyceraldehyde 3-phosphate dehydrogenase (*GAPDH*) and β-catenin [31].

**Table 3:** Primer sequences used for quantitative RT-PCR analyses

Gene name	Accession no.	Product size, bp	Direction	Sequence (5′→3′)
*PAX3*	NM_001206818.1	200	Forward	AGTGAGTTCCATCAGCCGCATC
			Reverse	TCTTCAGGGGCAAGTCTGGTTC
*PAX7*	XM_015462509.1	107	Forward	CGGGCATGTTTAGCTGGGAGA
			Reverse	TCTGAGCACTCGGCTAATCGAAC
*MYOD*	NM_001040478.2	111	Forward	GAACTGCTACGACCGCACTTACT
			Reverse	GAGATGCGCTCCACGATGCT
*MYF5*	XM_015470879.1	93	Forward	CCCACCAGCCCCACCTCAAGT
			Reverse	GTAGACGCTGTCAAAACTGCTGCT
*MYOG*	NM_001111325.1	131	Forward	CTCAACCAGGAGGAGCGCGAC
			Reverse	TTGGGGCCAAACTCCAGTGCG
*18S*	NR_036642.1	118	Forward	CCTGCGGCTTAATTTGACTC
			Reverse	AACTAAGAACGGCCATGCAC
*PGC1A*	NM_177945.3	200	Forward	TGATTAGTTGAGCCCTTGCCG
			Reverse	TGCCAGGAGTTTGGTTGTGAT

### Western blot analysis

Proteins were extracted from muscle tissue using lysis buffer (1% SDS, 10 mmol/L Tris-HCl, pH 8.0, 10 mmol/L NaCl, 3 mmol/L MgCl_2_, 0.5% NP-40, and 10 mmol/L NaF). Western blotting was performed as previously described [32]. The protein bands were visualized using the Odyssey Infrared Imaging System (LI-COR Biosciences, Lincoln, NE). The primary antibodies used were myogenin (F5D, Developmental Studies Hybridoma Bank, Iowa City, IA) and β-tubulin 90 (#2146, Cell Signaling, Danvers, MA). Protein band density was quantified and normalized to β-tubulin content.

### Statistical analysis

Unpaired t-test was performed to analyze the difference in means. All data were found to be normally distributed. Significance was accepted at *P* < 0.05. All data are expressed as means ± standard errors of the mean (SEM).

## Results

### Vitamin A administration upregulates the expression of myogenic genes

**Fig. 1:**
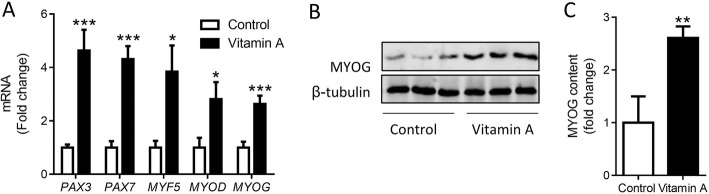
Vitamin A administration upregulates the expression of myogenic genes. **a** The level of myogenic mRNAs in muscle biopsy. **b** Protein content of MYOG in muscle biopsy. **c** Quantification of MYOG protein. *P < 0.05, **P < 0.01, ***P < 0.001; Mean ± SEM; *n* = 9

Our previously study showed that vitamin A administration at birth strongly increased beef cattle growth [28], which was mostly likely through promoting muscle growth because muscle accounts close to 50% of body weight and the hip length was not affected (data not shown). To explore the effects of vitamin A administration on myogenesis, we analyzed the expression of myogenic genes in muscle biopsy tissue obtained at 2 months of age. The expression of myogenic transcription factors including *PAX3*, *PAX7*, *MYF5*, *MYOD* and *MYOG* was enhanced due to vitamin A treatment (Fig. 1a). Consistently, vitamin A significantly increased MYOG protein content (Fig. 1b and c). These data demonstrated that vitamin A promoted myogenesis in calf muscle.

### Vitamin A injection increases density of satellite cells

**Fig. 2:**
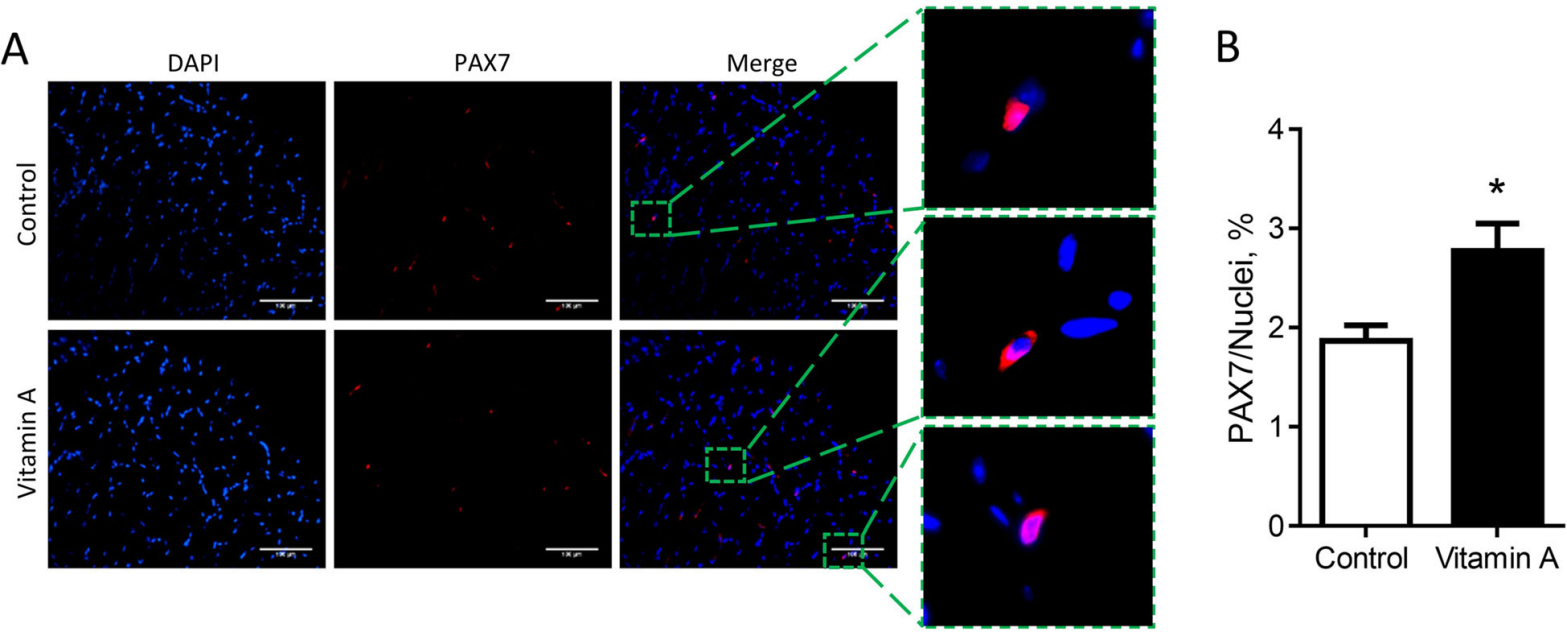
Satellite cell density is increased due to vitamin A injection. **a** Muscle biopsy stained by PAX7 (scale bar = 100 μm). **b** Quantification of PAX7^+^ cells in muscle biopsy. *P < 0.05; Mean ± SEM; *n* = 9

**Fig. 3:**
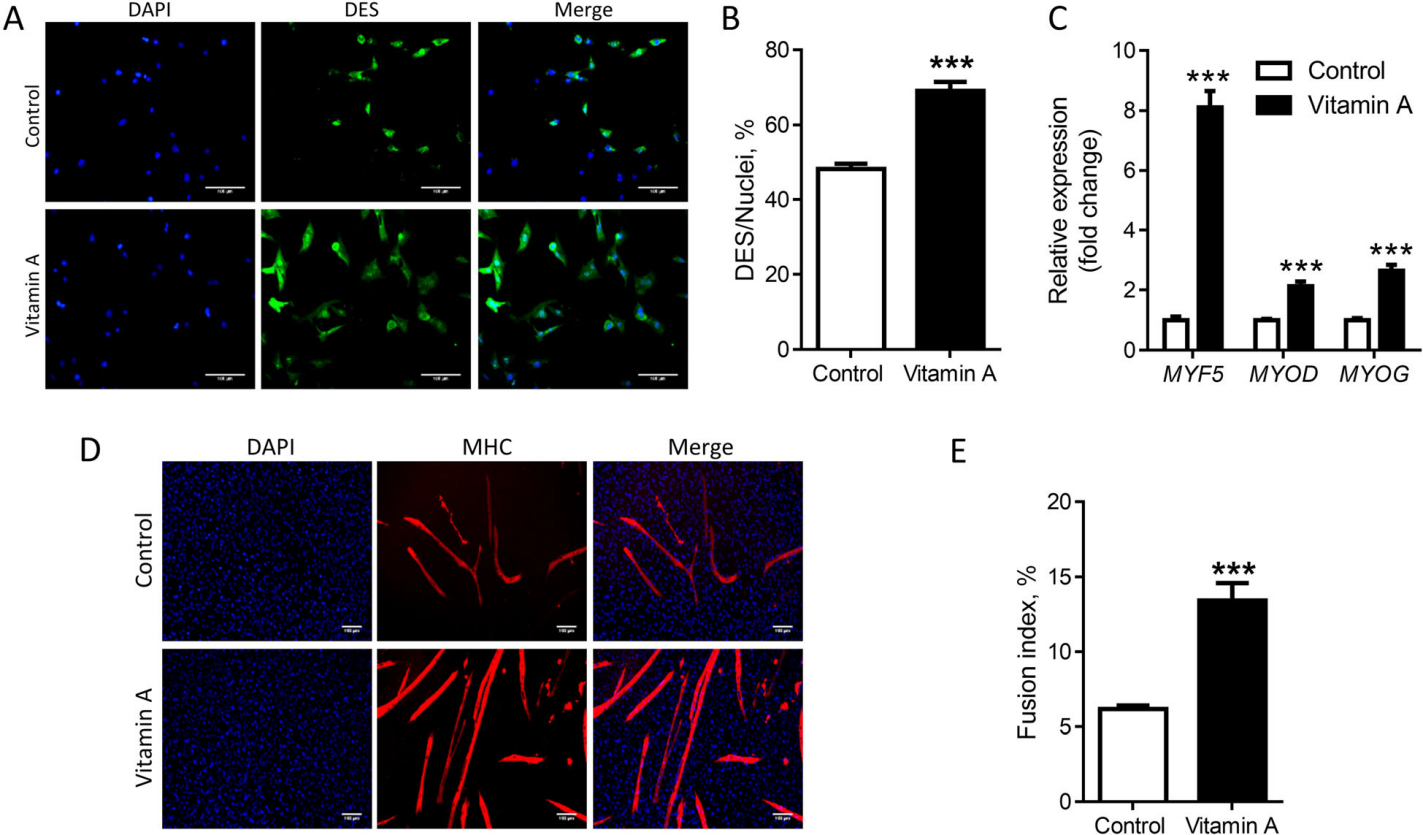
Myogenic differentiation of isolated mononuclear cells is increased in vitamin A treated calves. **a** Mononuclear cells isolated from muscle biopsy were stained with anti-Desmin antibody. **b** Quantification of Desmin positive cell number. **c** The level of myogenic mRNAs after 2 d of myogenesis. **d** Bright field pictures of cells before inducing myogenic differentiation (scale bar = 100 μm) and immunofluorescence staining of myotubes using anti-MHC antibody after 6 d of myogenic differentiation. **e** Fusion index. ***P < 0.001; Mean ± SEM; *n* = 9; scale bar = 100 μm

Using immunohistochemical staining of the muscle biopsy of 2-month old calves, we found that vitamin A injection increased PAX7^+^ satellite cells (Fig. 2a and b). In addition, we further separated muscle derived mononuclear cells which contain both myogenic satellite cells and nonmyogenic stromal vascular cells [30]. These cells were stained with the myogenic specific marker desmin immediately after the cells attached to the plates. Desmin staining was higher in mononuclear cells separated from vitamin A treated muscle biopsy (Fig. 3a and b). After 2 d of myogenic differentiation, the mRNA expression of *MYF5*, *MYOD* and *MYOG* was higher in the vitamin A group (Fig. 3c). After 6 d, cells were stained for the presence of terminal myogenic differentiation marker, myosin heavy chain (MHC), which expresses in fused myotubes. More myotubes were observed in the vitamin A group (fusion index, Fig. 3d and e), which were consistent with the higher myogenic population in mononuclear cells of vitamin A treated calves (Fig. 2a-b and Fig. 3a-b). Such enhanced myogenesis was associated with a higher population of satellite cells, as shown by increased PAX7 and desmin positive cells.

### Effects of neonatal vitamin A administration on beef cattle muscle size

**Fig. 4:**
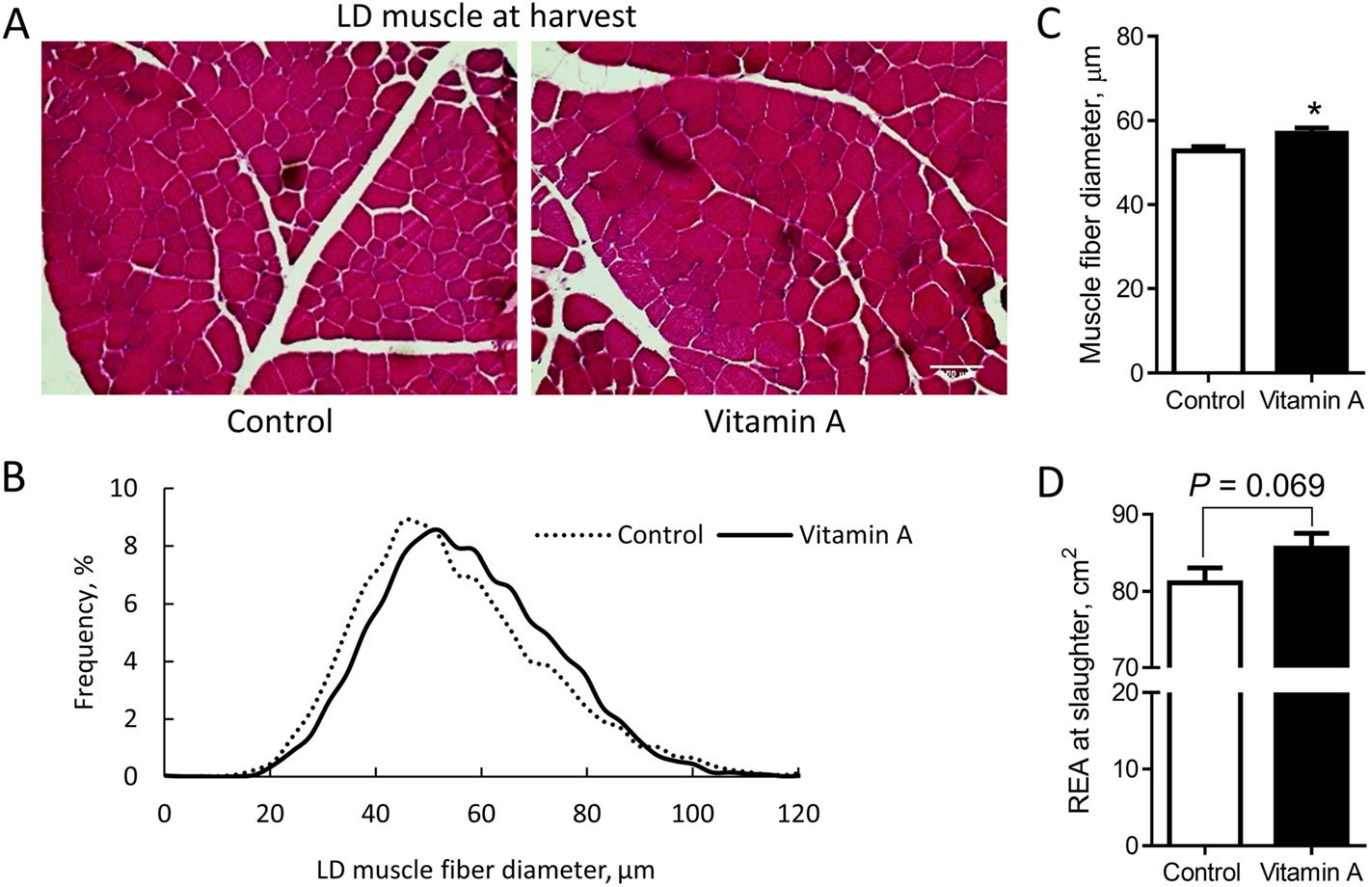
Muscle fiber diameter and distribution of calves were altered due to vitamin A treatment. **a** H&E stained LD muscle at harvest (scale bar = 100 μm). **b** Distribution of fiber diameter of LD muscle at harvest. **c** Average LD muscle fiber diameter at harvest. **d** Average REA at harvest. *P < 0.05; Mean ± SEM, *n* = 9

Consistent with the upregulation of myogenic genes and increased satellite cell density in biopsy samples of vitamin A treated calves, the average size of muscle fiber in vitamin A treated cattle at harvest was larger (Fig. 4a-c). In agreement, the REA was also higher (*P* = 0.069) in vitamin A treated cattle (Fig. 4d).

### Vitamin A injection increases oxidative muscle fibers

**Fig. 5:**
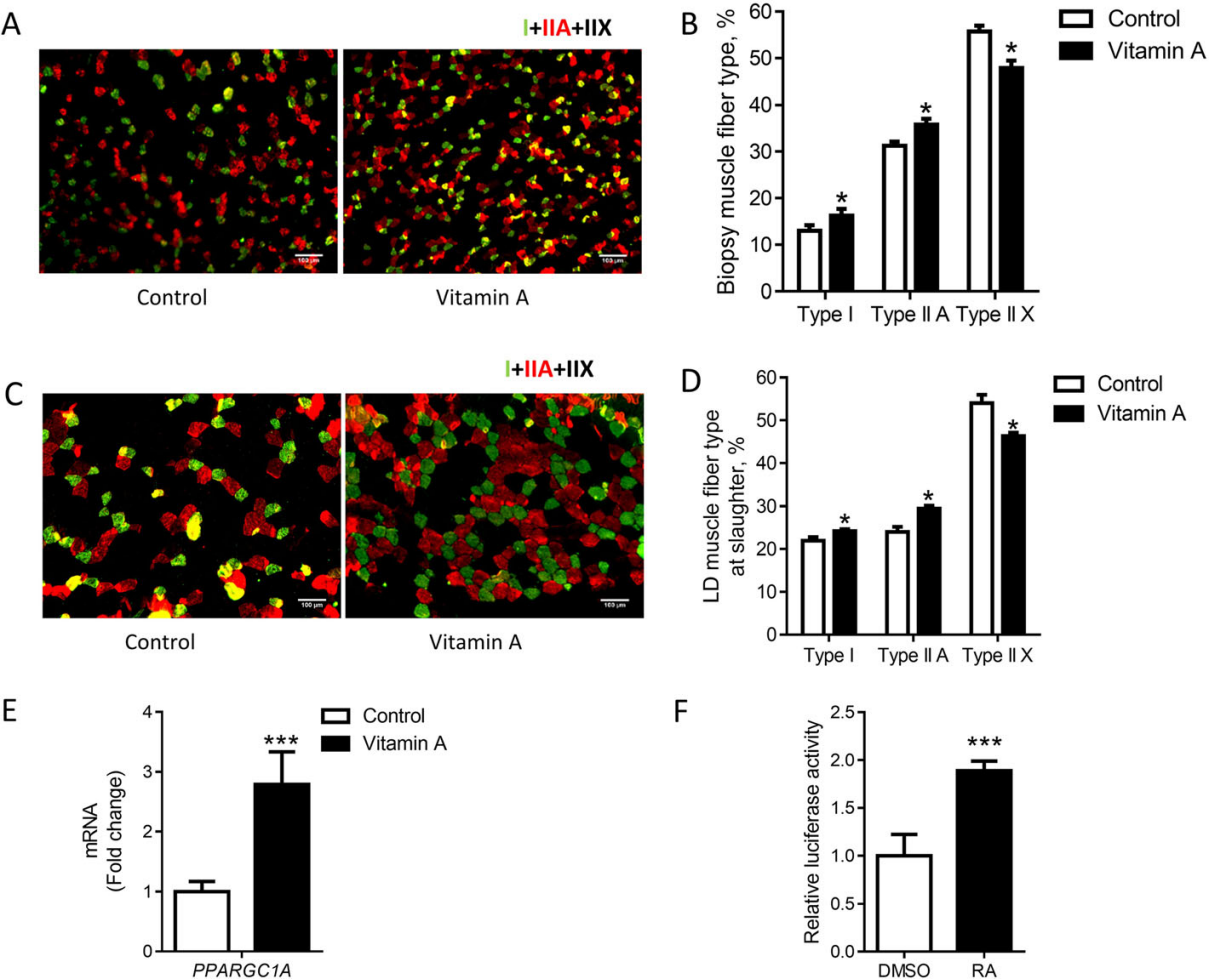
Vitamin A treatment increased oxidative muscle fibers. **a** Representative images showing fiber types in biopsy muscle (scale bar = 100 μm). **b** The proportion of fiber type composition in biopsy muscle. **c** Representative images showing fiber types in LD muscle at harvest. **d** The proportion of fiber type composition in LD muscle at harvest. **e** mRNA levels of *PPARGC1A* in biopsy muscle. **f** Mononuclear cells isolated from cattle muscle without vitamin A treatment were transfected with *Pgc1a* plasmid and treated with RA for 4 h, and the luciferase activity driven by the *Pgc1a* promoter was analyzed. *P < 0.05, ***P < 0.001; Mean ± SEM; *n* = 9

We further determined the composition of muscle fiber types in biopsy muscle by immunohistochemical staining. Vitamin A injection increased the portion of type I and type IIa oxidative muscle fibers, but reduced that of type IIX glycolytic muscle fibers in 2-month biopsy muscle (Fig. 5a and b). Consistently, vitamin A administration increased the portion of type I and type IIa oxidative muscle fibers, but reduced that of the type IIX glycolytic muscle fibers in LD muscle at harvest (Fig. 5c and d). We furtherly found that vitamin A injection upregulated the expression of *PPARGC1A* (Fig. 5e), a transcription factor that drives the formation of oxidative muscle fibers [14]. Consistently, the metabolite of vitamin A, all-trans RA, activated *PPARGC1A* promoter and promoted its transcription in cultured primary myogenic cells (Fig. 5f). There data showed that vitamin A administration increased oxidative muscle fibers which is likely through upregulation of *PPARGC1A* by RA.

## Discussion

Beef cattle and other meat animals are raised primarily for their muscle and, thus, enhancing muscle growth is critical for improving production efficiency. Muscle growth is due to both myofiber hyperplasia and hypertrophy. The number of muscle fibers is determined primarily before birth in livestock species [33–35]. During fetal development, muscle fibers are formed through primary and secondary myogenesis. A portion of myogenic cells become quiescent and form the stem cell pool for later myogenesis; these stem cells are termed satellite cells [36]. During rapid muscle growth and under injury postnatally, satellite cells are activated and proliferated; a portion of daughter cells replenish the stem cell pool while the majority fuse with existing muscle fibers to increase muscle fiber sizes or generate new muscle fibers to replace damaged fibers [37].

RA is an important morphogen during embryonic and fetal development [21]. Dietary vitamin A is converted into RA, which serves as a ligand for RAR [19], and form a complex with RXR to bind to RAR response elements (RAREs) on target genes [20]. Two cellular retinoic acid binding proteins regulate RA partitioning, with cellular RA binding protein II (CRABP-II) delivers RA to RARs, and fatty acid binding protein type 5 (FABP5) to PPARβ/δ [38–40], generating different biological effects [41]. A large number of studies have been conducted on the role of RA in lipid metabolism in beef cattle [42–49]. In comparison, the role of vitamin A in muscle growth has only been sparsely studied. To the knowledge of authors, no study was conducted on the role of RA in myogenesis in beef cattle. Based on studies on other species, RA plays an important role in myogenic lineage commitment through binding to RXR [50, 51]. RA blocks chondrogenesis and stimulates myogenesis of limb bud mesenchymal cells [52]. RA promotes in vitro formation of pre-myogenic mesoderm and upregulates myogenic precursor genes [53]. RA is required not only for the myogenic precursor cell commitment but also for the later myogenic differentiation. RA signaling maintains Pax3 and Meox2 in the progenitor and Myf5 and MyoD in the differentiating myoblasts [54]. In the early stage of limb bud development, RA recruits muscle progenitor cells into the limb [54]. RA promotes myogenesis by antagonizing TGFβ signaling via inhibiting C/EBPβ [55] as well as activating FGF8 signaling [26].

In agreement with enhanced myogenesis, in the current study, we found that neonatal vitamin A injection increased the population of satellite cells. Satellite cell proliferation and fusion with existing muscle fibers are critical for postnatal growth of meat animals [56]. The increased satellite cells due to vitamin A administration at the early stage contributed to muscle growth later. Consistently, the average size of muscle fibers was higher in vitamin A treated cattle compared to control cattle. In addition, the REA was larger (*P* = 0.069) in treated cattle, showing the long-term effect of early vitamin A treatment in the muscle growth of beef cattle. The increased muscle growth also explained the increase in body weight observed in the previous study [28], where vitamin A administration significantly increased body weight without influencing bone growth, suggesting an increase in muscle mass.

Muscle fiber type composition affects the quality of meat [57]. PPARGC1A signaling plays an important role in the development of oxidative muscle fibers via regulating mitochondrial functional capacity and cellular energy metabolism [58, 59]. Forced overexpression of *PPARGC1A* in skeletal muscle increases oxidative type IIA and type I fibers [14]. On the contrary, muscle specific *PPARGC1A* knock out mice exhibited a shift from type IIA and type I toward type IIB and IIX muscle fibers [60]. *PPARGC1A* is inducible in response to increased mitochondrial energy demand in different physiologic conditions, such as exercise [61, 62] and fasting [63]. PPARGC1A mediates exercise induced adaptation of muscle fibers [62, 64]. In skeletal muscle, PPARGC1A is found to be activated by AMP-activated protein kinase (AMPK), a key regulator of cellular energy homeostasis [65]. RA increased *PPARGC1A* expression in brown adipocytes [66]. Consistently, we found that RA upregulated *PPARGC1A* by directly activating its promoter, which in turn increased oxidative muscle fibers. Our data are in agreement with a previous report in mice where RA was found to stimulate oxidative capacity of muscle via activation of PPARGC1A [67]. In summary, vitamin A administration increased the proportion of oxidative muscle fibers and slightly reduced that of glycolytic muscle fibers. The slight change in muscle fiber composition due to vitamin A treatment in calves might improve the water holding capacity of beef through reducing postmortem glycolysis and thus the ultimate pH of beef. Also, oxidative muscle fibers are associated with higher amounts of intramuscular fat and intramyocellular lipids, which may also improve beef quality.

## Conclusion

Vitamin A administration to neonatal calves increased muscle growth, which is associated with enhanced satellite cell activation. In addition, vitamin A treatment during the early stage had long-term effect on muscle fiber type composition in beef cattle by shifting to oxidative fiber types. Thus, to obtain optimal growth performance, the vitamin A levels in beef cattle need to be carefully managed.

## Abbreviations

LD: latissimus dorsi
MHC: myosin heavy chain
MYOD: myogenic differentiation 1
MYOG: myogenin
NIR: near-infrared spectroscopy
PAX7: paired box 7
PFA: paraformaldehyde
PPARGC1A: PPARG coactivator 1 alpha
RA: retinoic acid
REA: rib eye area
